# Iterative selection of lipid nanoparticle vaccine adjuvants for rapid elicitation of tumoricidal CD8⁺ T cells

**DOI:** 10.1016/j.bioactmat.2025.01.028

**Published:** 2025-02-18

**Authors:** Yuan Luo, Shiqi Zhou, Yiting Song, Wei-Chiao Huang, Gregory E. Wilding, James Jablonski, Breandan Quinn, Jonathan F. Lovell

**Affiliations:** a Department of Biomedical Engineering, State University of New York (SUNY) at Buffalo, Buffalo, NY, 14260, USA; b Department of Biostatistics, SUNY at Buffalo, Buffalo, NY, 14214, USA

## Abstract

A challenge for cancer vaccines is to elicit immune responses of sufficient magnitude to control malignant tumor growth and spread. In this study, we iteratively screened a panel of 22 lipid-phase vaccine adjuvants in mice for the elicitation of neoantigen-specific CD8⁺ T cell responses, using an integrated peptide-lipid nanoparticle approach. CL401, a dual Toll-like receptor 2/7 (TLR2/7) adjuvant rapidly induced neoantigen-specific T cell responses and improved lymphatic drainage and uptake of the particle. Additional rounds of *in vivo* screening identified complementary adjuvants which targeted TLR4 (3D6A-PHAD adjuvant), TLR8 (motolimod), and inflammasome (QS-21) pathways and synergized to enhance cytokine secretion in antigen presenting cells and vaccine-elicited neoantigen-specific CD8⁺ T cells. Co-delivery of adjuvants and antigens led to effective immune responses which regressed large established tumors, synergized with immune checkpoint blockade, and inhibited lung nodules in an experimental metastasis model, without overt toxicity or reactogenicity. We conclude that iterative adjuvant screening, performed in mice *in vivo*, can identify useful adjuvant combinations that hold potential for therapeutic cancer vaccine research.

## Highlights

• 22 vaccine adjuvants were screened *in vivo* using a neoantigen-lipid nanoparticle system.
• Iterative screening identified adjuvants combinations to induce strong neoantigen responses.
• Vaccination with the neoantigen and adjuvant combination led to anti-tumor responses in mice.

## Graphical abstract

**Figure:**
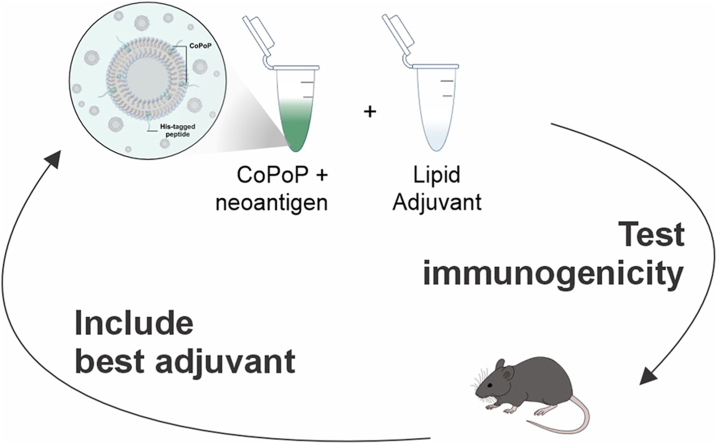

## 1 Introduction

Cancer immunotherapy has had profound impact on the practice of oncology. It involves the administration, elicitation, or unleashing of effector cells that recognize and kill cancer cells [1]. One area of immunotherapy where nanoscale approaches have generated interest is cancer vaccines [2]. The efficacy of such vaccines benefits from the ability of nanomaterials to serve as delivery vehicles and to direct immunomodulatory agents to lymphoid organs [3]. Therapeutic cancer vaccines generally aim to induce cytotoxic CD8^+^ T cells to recognize and lyse tumor cells displaying tumor-specific or tumor-associated antigens [4,5]. Despite broad efforts, therapeutic cancer vaccines have traditionally struggled to show encouraging responses in clinical trials [6], as elicited T cells may face challenges with insufficient frequency, poor tumor infiltration, an immunosuppressive tumor microenvironment, or escape or downregulation of displayed tumor antigens. However, there has been a renewed interest in the utilization of cancer vaccines, especially based on mRNA, directed against personalized, tumor-specific neoepitopes [7].

To deliver antigens, cancer vaccines have utilized peptides [8,9], nucleic acids [10,11] and viral vectors [12,13]. Peptides are low-cost, simple to manufacture and have an appealing safety profile. Generally, these peptides contain one or multiple epitopes derived from tumor specific or associated antigens but often lack sufficient antigenicity on their own. Therefore, they are more effective when administered in combination with adjuvants [[14], [15], [16]]. Toll-Like receptors (TLRs) are a family of well-studied and structured pattern recognition receptors. TLR 1, 2, 4, 5, 6 are more expressed on cell surfaces whereas TLR 3, 7, 8, 9 are more expressed in intracellular compartments like endosomes [17]. When their agonists (e.g., several types of vaccine adjuvants) are delivered, downstream transcription factors are activated, ultimately leading to cytokine secretion, which in turn activates surrounding cells and shapes the immune response [[18], [19], [20]]. Individual TLR agonists can induce varying cytokine profiles depending on their administration and target cells, leading to immunostimulatory but possibly even immunosuppressive effects [21]. Emerging lines of research suggest that multiple adjuvants can induce stronger synergistic anti-tumor effects [[22], [23], [24], [25]]. Lipid nanoparticles have attracted attention as vaccine platforms and studies suggest that they can also benefit from adjuvant inclusion [26,27] and lipid nanoparticle design [28,29].

Herein, we report an iterative *in vivo* vaccine adjuvant screening and selection approach to identify a multiple adjuvant combination for potent cancer vaccines. Iterative selection involved multiple rounds of immunization wherein groups of mice were immunized with a different adjuvant, and then the immunogenic adjuvants were retained in the vaccine system in the next round of screening for multiple rounds. To do this, we utilized liposomes containing cobalt porphyrin–phospholipid (CoPoP), which have previously been shown to be an effective delivery technology for short peptides cancer vaccines [30,31] and the technology has progressed through human clinical trials for a COVID-19 vaccine [32,33]. Modification of the peptides with three histidine residues enables peptide conversion into liposome-bound format upon interaction with CoPoP, leading to antigens forming a depot at the injection site and the co-delivery of antigens and adjuvants. CoPoP liposomes have previously been used for *in vivo* screening to identify a short murine cancer CD8^+^ neoepitope termed Nes2LR fom the Renca tumor cell line [34] and to identify tumor-associated antigens with mutations that lead to improved functional immunogenicity. However, which immunostimulatory adjuvants are optimal with this particular delivery system for preclinical studies in mice has not been addressed. To date, the adjuvants of monophosphoryl lipid A (MPLA) and the saponin (QS-21) have been used, which are also components of the clinically used AS01 vaccine adjuvant system [35]. Using our recently identified short, H2K^d^-restricted, tumor-specific neoantigen [34], we present the results of iterative *in vivo* adjuvant screening using CoPoP-neoantigen particles with integrated lipid adjuvants to identify adjuvants that can rapidly induce antigen-specific CD8^+^ T cell responses and which additional adjuvants can be added for further immunogenic synergy.

## 2 Results

### 2.1 CL401 synergizes with motolimod, 3D6A-PHAD and QS-21 for neoantigen vaccine responses in mice

For adjuvant screening, we used an iterative process to find an optimal combination. 22 commercially available lipid-phase immunostimulatory adjuvants (listed in Table S1 in the Supplementary Information) that should theoretically incorporate into lipid bilayers and operated on diverse mechanisms were selected for screening. To determine adjuvant efficacy, we assessed antigen-specific IFN-γ responses in post-vaccinated mouse T cells with antigen restimulation. In general, the adjuvant that induced the highest immune response in each round was then incorporated into the CoPoP liposome and used as a basis for combination with other adjuvants in next round. Then, the most effective combination of two adjuvants were selected as the new basis for the further rounds of screening. Finally, through the iterative process, an optimal adjuvant combination for CoPoP liposomes was identified. In the initial screen, adjuvants were dissolved in ethanolic solution and individually incubated with lipid nanoparticles preloaded with CoPoP and the short Nes2LR 9mer neoantigen derived from the Renca cancer cell line. The *Quillaja Saponaria* (QS) saponin adjuvants were added from aqueous solution as they are expected to bind to the cholesterol included in the bilayer [36]. The addition of each adjuvant did not impact particle size, which remained around 150 nm (Supplemental Fig. S1A). A fluorescence quenching assay suggested that the Nes2LR peptide bound to all adjuvanted CoPoP liposomes without exception, but not to control liposomes lacking CoPoP but containing the cobalt-free porphyrin-phospholipid (PoP) (Supplemental Fig. S1B).

**Fig. 1:**
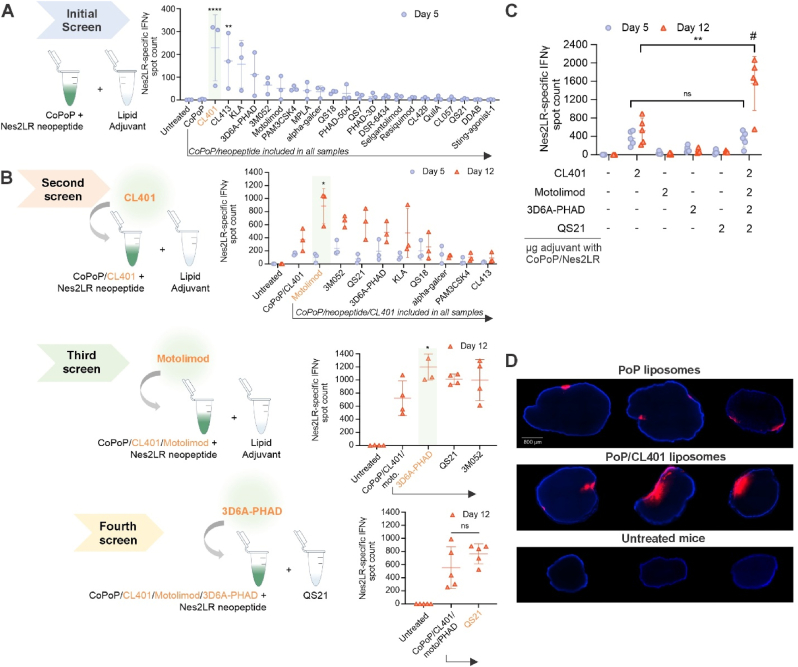
**Iterative adjuvant screening*****in vivo*****reveals that CL401 rapidly elicits neoantigen-specific CD8**^**+**^**T cells with vaccination, while Motolimod, PHAD, and QS-21 enhance boosting**. **A**, Initial screen: CoPoP/Nes2LR particles were incubated with or without each adjuvant. BALB/c mice were immunized on day 0 (2 μg adjuvant per mouse) and PBMCs were re-stimulated on day 5 asses IFN-γ spots by ELIspot. **B**, Additional iterative screening steps were carried out in a similar manner, with a “base” formulation that incorporates the best performing adjuvant from the prior screen. In the second round: CL401/CoPoP/Nes2LR was incubated with or without other effective adjuvants. Mice were immunized on day 0 and day 7 (2 μg adjuvant per mouse). Blood was collected on day 5 and day 12 for IFN-γ ELIspot analysis. The previous steps were repeated for rounds three and four. Statistical comparison based on one-way ANOVA with Dunnett’s multiple comparison with the base vaccine group from where the arrow emanates. **C**, IFN-γ secretion with various adjuvant compositions. Mice were immunized with various vaccine formulations on days 0 and 7, with the adjuvant doses for each group indicated. **D**, Fluorescence micrographs showing uptake of lipid nanoparticles in draining lymph nodes. Mice were immunized with PoP or PoP/CL401 liposomes (2 μg CL401 per mouse) on day 0. Inguinal lymph nodes were collected 24 h later and cryosectioned for imaging. Blue represents DAPI while the red shows the liposomal PoP. Data shows mean ± SD with n = 3 biological replicates (**A**), n = 3,4,5 (**B**), n = 5 (**C**), n = 3 (**D**), and analyzed by oneway ANOVA with Dunnett’s multiple comparisons test. ∗*p* < 0.05, ∗∗*p* < 0.01, ∗∗∗*p* < 0.001, ∗∗∗∗*p* < 0.0001. # represents significance of synergism of C27a compared to the contribution of all individual adjuvants on day 12 (P = 0.018) as defined in the methods section based on a linear contrast with a fitted ANOVA model.

Adjuvanted CoPoP liposomes were then used to vaccinate mice and Nes2LR neoantigen-specific CD8^+^ T cells were assessed shortly (5 days) after the initial immunization (Fig. 1A). Without adjuvant inclusion, no neoantigen-specific T cells were induced, as the ELIspot count number was similar to that of untreated mice. Antigen-specific CD8^+^ T cell elicitation was assessed by measuring IFN-γ secretion in short peptide neoantigen-restimulated peripheral blood mononuclear cells (PBMCs). Among the adjuvant panel, CL401, a dual TLR2/7 agonist, stood out for inducing a high frequency of neoantigen-specific T cells. CL413, a structurally similar dual TLR2/7 agonist also rapidly elicited a neoantigen-specific cellular immune response. Tetramer staining confirmed these results, confirming the elicitation of neoantigen-specific CD8^+^ T cells (Supplemental Fig. S1C).

Since CL401 rapidly induced neoantigen-specific CD8^+^ T cells upon integration into neoantigen peptide particles, we next repeated the *in vivo* adjuvant screening process, but this time with incorporation of CL401 into all vaccines for screening (along with CoPoP and the Nes2LR neoantigen) to determine if the inclusion of a second adjuvant could further benefit immune responses (Fig. 1B). In the second screening round, the “alone” group refers to the CL401/CoPoP/Nes2LR. All the vaccines were administered on day 0 and 7. Blood samples were collected on day 5 and 12. While no other adjuvants enhanced the pre-boost response, the combination of the TLR8 agonist Motolimod with CL401 showed enhanced immunogenicity following the boost compared to lipid particles adjuvanted with CL401 alone. This adjuvant selection and screening process was repeated a third time, and in the next round, the TLR4 agonist 3D6A-PHAD was found to enhance the cellular immunogenicity of lipid nanoparticles that also contained Motolimod and CL401. Finally, the saponin adjuvant QS-21 was assessed in the final round of screening as it had consistently increased cellular responses in prior screenings with CL401. The addition of QS-21 to lipid nanoparticles containing CL401, Motolimod, and 3D6A-PHAD led to higher immunogenicity, although the increase was not statistically significant. Nevertheless, QS-21 was included into the formulation as it is an adjuvant that has been shown to be effective in human vaccines. As CL401 is the key adjuvant identified for rapid responses and is a TRL 2/7 agonist, and the other adjuvants operated on TRL 4, TRL, 8 and inflammasome pathways, we termed this formulation as 2/7 adjuvanted, or 27a. With CoPoP inclusion for short peptide integration, this adjuvant system is referred to here as C27a. Importantly, the inclusion of all tested adjuvants did not impact the liposomes particle size (Supplemental Fig. S2A) or peptide binding efficiency (Supplemental Fig. S2B). Furthermore, the frequency of neoantigen-specific CD8^+^ T cells was consistent with that of IFN-γ result (Supplemental Fig. S2C). To interrogate the contribution of each adjuvant in the C27a system, mice received vaccines containing each individual adjuvant alone or the complete 27a formulation containing all 4 adjuvants. Results from IFN-γ ELIspot (Fig. 1C) showed that CL401 and C27a induced the highest antigen-specific IFN-γ spot counts on day 5, consistent with the initial screening result. The second immunization showed a strong boosting effect only in the C27a group, but not in the CL401 alone group. When administered as the only adjuvant in the integrated lipid nanoparticles, Motolimod, 3D6A-PHAD, and QS-21 were unable to effectively induce a substantial frequency of neoantigen-specific CD8^+^ T cells even after boosting. However, when co-administrated in the form of C27a, the frequency of neoantigen-specific CD8^+^ T cells increased drastically. Compared to all other groups baring single antigens, the combination of the 4 adjuvants in the C27a vaccine exhibited significant synergism (P = 0.03, indicated by “#” symbol). The improved immunogenicity of C27a was also confirmed based on neoantigen-specific CD8^+^ tetramer staining (Supplemental Fig. S3A).

### 2.2 CL401 induced rapid immune responses with improved delivery to dendritic cells

To explore the mechanisms underlying the rapid adjuvant activity of CL401, liposomes containing PoP as a fluorescent indicator were used to assess the vaccine uptake in antigen presenting cells. *In vitro,* RAW264.7 cultures showed that within a short period of 3 h, cells took up PoP liposomes (Supplemental Figs. S3B and C). *In vivo,* following intramuscular injection, the inclusion of CL401 significantly enhanced the uptake in draining inguinal lymph nodes (dLN), which were collected 24 h after immunization. Fluorescence images showed a higher accumulation of PoP fluorescence in the PoP/CL401 group (Fig. 1D). Flow cytometry was also performed to quantitatively assess liposome uptake in lymph nodes (Supplemental Fig. S4A). PoP liposomes facilitated the recruitment of a higher number of antigen presenting cells (APCs) residing in the lymph nodes. CL401 inclusion resulted in twice the liposome uptake within the lymph nodes, and notably within dendritic cells (DCs), which likely contributed to the rapid immune response (Supplemental Fig. S4B). To further investigate the mechanism of enhanced CL401 uptake in lymph nodes, we investigated the activation of TLR2 signaling pathway. CL401 was found to specifically stimulate TLR2. This signal was increased twofold when CL401 was formulated in CoPoP liposomes. Additionally, the strongest activation observed in the C27a group indicated that the adjuvants combination against multiple receptors may lead to the cascade amplification of signaling pathways (Supplemental Fig. S4C).

### 2.3 Combined adjuvants in C27a enhance upstream and neoantigen-specific downstream cytokine secretion

**Fig. 2:**
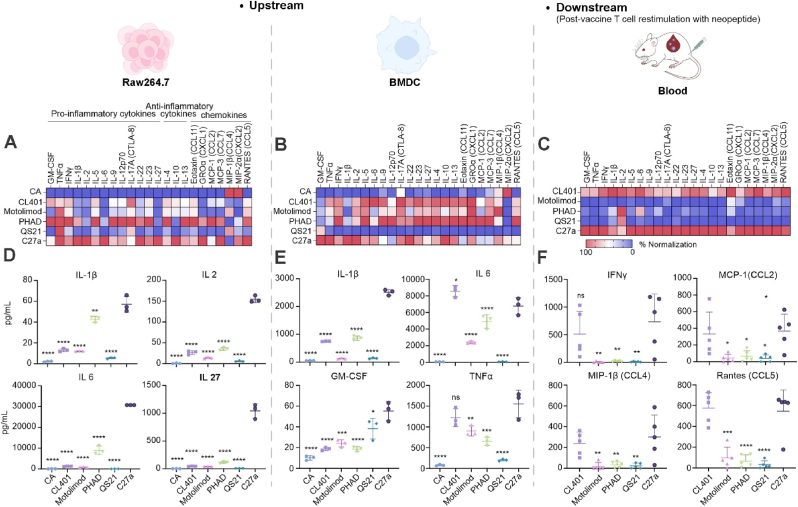
**C27a activates upstream and antigen-specific downstream cytokine secretion.** Heatmap of induced cytokines secreted by RAW264.7 (**A**), BMDCs (**B**) and Nes2LR neoantigen-restimulated, post-vaccinated T cells (**C**). RAW264.7 and BMDCs were treated with 10 μg/mL each adjuvant for 24 h at 37 °C. Nes2LR neoantigen-specific T cells were obtained on day 14 from the blood of mice immunized twice with (2 μg antigens and 2 μg each adjuvant per mouse). Each square represents the average of n = 3 (**A-B**), n = 5 (**C**) biological replicates. All the data are plotted by average normalized. RAW264.7 (**D**), BMDCs (**E**) and neoantigen-specific T cells (**F**) quantitative analysis of selected secreted cytokines. Data show mean ± SD with n = 3 (**D-E**), n = 5 (**F**) and analyzed by ordinary one-way ANOVA with Turkey’s multiple comparisons test. ∗*p* < 0.05, ∗∗*p* < 0.01, ∗∗∗*p* < 0.001, ∗∗∗∗*p* < 0.0001.

As C27a contains CL401 (dual TLR2/7 agonist), 3D6A-PHAD (TLR4), motolimod (TLR8), and QS-21 (inflammasome) against different receptors, we investigated the cytokine and chemokine secretion levels of immune cells after stimulation to exhibit the diversified ways that these adjuvants impacted immune response. Macrophages and dendritic cells (DCs) are two key antigen-presenting cells and play crucial roles in initiating immune responses. RAW264.7 and bone marrow-derived dendritic cells (BMDCs), utilized as an "upstream" immune model of initial vaccination response, were stimulated *in vitro* by adjuvants. Generally, cytokine titers increased with increasing adjuvant concentrations, usually reaching a peak at 10 μg/mL, whereas chemokine levels exhibited an opposite trend. CCL11, MCP-1, MCP-3 reached the peak at 0.625 μg/mL. IL6 and IL27 secreted by RAW264.7 within 24 h reached a high level even at the low dose of C27a (Supplemental Fig. S5). When exploring the effect of time course on cytokine secretion, we found that after RAW cells were stimulated with adjuvants, almost all cytokines and chemokines could be accumulated and released within 24 h (Supplemental Fig. S6). Therefore, we focused on comparing the cytokine and chemokine secretion by RAW264.7 and BMDCs after stimulation with different adjuvants for 24 h. C27a synergistically augmented the secretion of IL-1β, IL2, IL6, IL27, CXCL1 in mouse RAW264.7 (Fig. 2A and D), as well as IL-1β, GM-CSF, TNFα in BMDCs (Fig. 2B and E) compared to each single adjuvant group. Among these, IL-1β, IL2, IL6 play critical roles in activating immune cells and amplifying proinflammation responses, while TNFα and IL27 can directly inhibit the growth of tumor cells. The enhanced secretion of IL10 caused by C27a, it can help regulate the excessive immune response. The secretion levels of other factors are shown in Table S3.

When the "downstream", neoantigen-specific T cells from the blood of vaccinated mice were restimulated by the same peptides, various cytokine and chemokine secretion levels indicated a robust T cell immune response elicited by C27a *in vivo* (Fig. 2C and F). A broad degree of cytokines were secreted upon antigen restimulation, including nearly every measured cytokine and chemokine on the panel. Even though other adjuvants, such as PHAD, had stimulatory effects on upstream APCs *in vitro* as well, when administrated alone in mice, there was a lack of strong cytokine-secreting T cell activation, with the exception of CL401.

### 2.4 Co-localization of adjuvants and antigens enhances immune responses

**Fig. 3:**
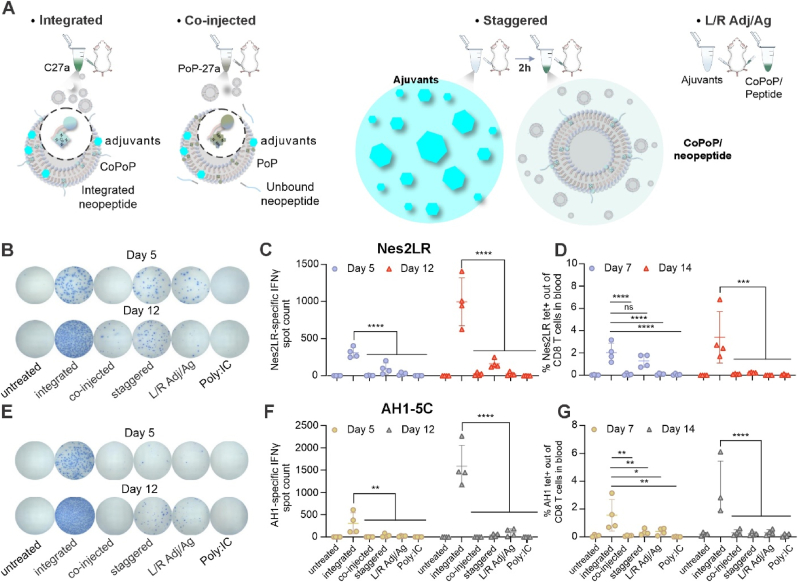
**Co-delivery adjuvants and neoantigens in integrated particles leads to strong neoantigen-specific T cell responses. A,** Scheme for vaccine preparation and administration routes. **B-D,** the immune response induced by Nes2LR epitope. Mice were immunized on day 0 and 7 (2 μg antigens per mouse). Blood was collected on day 5 and day 12 for ELIspot, day 7 and day 14 for tetramer staining. IFN-γ ELIspot results (**B**), quantitative analysis of IFN-γ spots (**C**) and flow cytometry result of Nes2LR tetramer (**D**). **E-G,** the immune response induced by AH1-5C epitope. The IFN-γ Elispot image (**E**), quantitative analysis of IFN-γ spots (**F**) and flow cytometry result of AH1 tetramer (**G**). Data was shown as mean ± SD with n = 4 (**B-G**) and analyzed by ordinary one-way ANOVA with Dunnett’s multiple comparisons test. ∗*p* < 0.05, ∗∗*p* < 0.01, ∗∗∗*p* < 0.001, ∗∗∗∗*p* < 0.0001.

To investigate the effect of temporal and spatial colocalization of adjuvants and antigens in eliciting immune responses, mice were vaccinated with the following four distinct dosing regimens: integrated, co-injection, staggered, and left/right (L/R) Adj/Ag. In the first two methods, the entire vaccine was injected into one leg of the mouse. For example, in integrated route, mice were received the complete C27a vaccine, containing four adjuvants, CoPoP liposome and neoantigen. In the co-injected route, the PoP-27a vaccines were administered, wherein CoPoP was replaced with PoP to prevent the binding of histidine-tagged peptides on the liposome surface. Consequently, peptides were co-injected with PoP liposomes into mice in their unbound state. However, in the staggered, and L/R Adj/Ag methods, CoPoP/peptides and adjuvants were injected separately. In the staggered method, mice were initially immunized with all adjuvants in the left leg, followed by injection of CoPoP/peptide liposomes at the same site after a 2-h interval. Finally, in the L/R Adj/Ag, adjuvants and CoPoP/peptides were simultaneously injected into two separate legs (Fig. 3A). Poly:IC as the common cancer vaccine adjuvant mixed with peptides was also included. IFN-γ ELIspot and tetramer staining in blood were performed on day 14. When adjuvants and Nes2LR were administered at different times and locations, there was a significant reduction in both the capacity of T cells to secrete IFN-γ in response to antigen re-stimulation (Fig. 3B and C) and the frequency of antigen-specific CD8 T cells (Fig. 3D). This pattern was consistently observed with another murine cancer epitope with a separate major histocompatibility complex class I (MHC-I) haplotype AH1-5C (H2Ld) (Fig. 3E–G). These data highlighted the advantage of co-localizing adjuvants and peptide epitopes in both time and space to elicit an optimal neoantigen-specific CD8^+^ T cell responses. Furthermore, the lack of T cell responses in the PoP-27a group that lacked cobalt (and therefore peptide display) illustrates the critical role of integrating short peptides into liposomes. This integration enhances the uptake by APCs and augmented the potential for epitopes to be presented MHC-I, which is requisite for an effective T cell immune response.

### 2.5 Anti-tumor responses

**Fig. 4:**
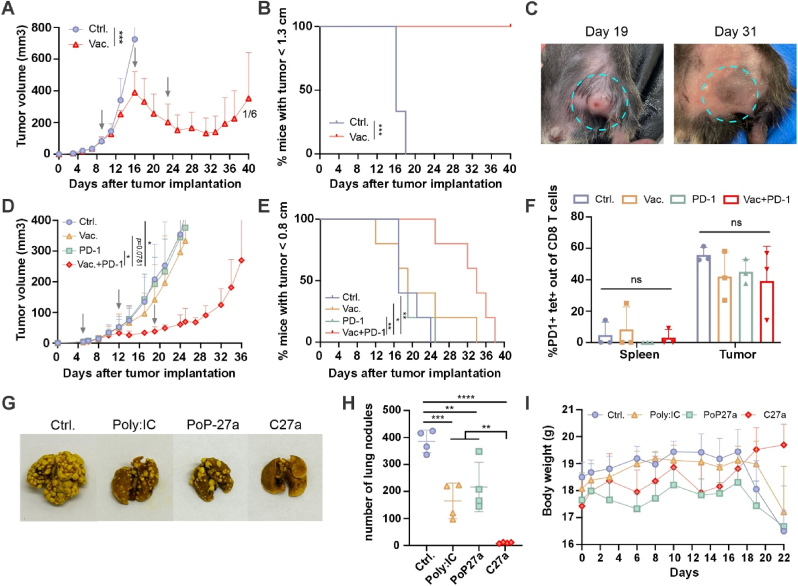
**C27a vaccination reverses large TC-1 tumor growth, synergizes with immune checkpoint blockade and inhibits a lung metastasis model.A**, TC-1 tumor growth. Mice were immunized C27a/E7_49-57_ vaccine (5 μg antigen, 10 μg CL401, 2 μg Motolimod, 2 μg 3D6A-PHAD, 2 μg QS-21 per mouse) on day 9, 16, and 23. **B**, The percentage of mice bearing with TC-1 tumors with length <1.3 cm. **C**, Photographs of an advanced TC-1 tumor (encircled) of the same mouse from the vaccine group on day 19 and day 31, reflecting tumor regression. **D**, Renca tumor growth curve in mice receiving the combination therapy of C27a vaccine and immune checkpoint blockade. Mice were immunized C27a/Nes2LR vaccine (5 μg antigen, 10 μg CL401, 2 μg Motolimod, 2 μg 3D6A-PHAD, 2 μg QS-21 per mouse) on day 5, 12, and 19. 100 μg Anti-PD1 antibody was administered on day 7, 9, 14, 16. **E**, The percentage of mice bearing with Renca tumors with length <0.8 cm. **F**, Expression of PD-1 on Nes2LR tet + CD8^+^ T cells in spleens and tumors. **G**, Pictures of lungs on day 22 harvested from mice untreated or treated with Poly:IC/Nes2LR, PoP-27a/Nes2LR, C27a/Nes2LR vaccines (n = 4). **H**, The quantitative analysis of nodules number. **I**, The body weight of mice with lung metastasis. Data was shown as mean ± SD (dots represent average of n = 6 (**A**), n = 5 (**D**), and n = 4 (**I**) and analyzed by unpaired *t*-test (**A,** the significance indicated the data of untreated group day 16 vs that of vaccine group day 25) or Log-rank (Mantel-Cox) test (**B, E**) or ordinary one-way ANOVA with Dunnett’s multiple comparisons test (**D, F, H,** the significance in **D** was compared the tumor volume data on day 25). ∗*p* < 0.05, ∗∗*p* < 0.01, ∗∗∗*p* < 0.001, ∗∗∗∗*p* < 0.0001.

To assess the efficacy of C27a as a cancer vaccine, a rapidly growing subcutaneous TC-1 tumor model was established on the right flank of C57Bl/6 mice. Compared to the untreated group, vaccinated mice showed significantly slower tumor growth. By the 16th day when the booster injection was given, the tumor volume of the untreated group had reached the end point of approximately 800 mm^3^, while the tumor volume in the vaccine group was controlled at half of that, about 400 mm^3^. Tumors began to regress immediately following the second vaccination, but finally relapsed on day 31 (Fig. 4A). The C27a/E7_49-57_ vaccine significantly improved survival (Fig. 4B), and one mouse had complete tumor regression (Fig. 4C). Photographs of other tumors in the C27a group before and after treatment are shown in Supplemental Fig. S7.

The potential of synergistic effect of anti PD-1 antibody and C27a/Nes2LR vaccine was assessed in the Renca tumor model. Nes2LR/C27a vaccines were administered on day 5 when tumors became palpable. Anti PD-1 antibodies were intraperitoneally injected on day 7, 9, 14, 16. Tumor volume regressed after the second dose on both vaccine group and combination group. On day 25, there was a significance on tumor volume between combination group and untreated or anti-PD-1 group (Fig. 4D). Moreover, the combination of vaccine and immune checkpoint blockade synergized improved the survival rate of mice bearing with Renca tumor (Fig. 4E). Tumor microenvironment analysis showed that PD-1 was highly expressed on neoantigen-specific CD8^+^ T cells in the tumor compared to the spleen, thereby providing strong rationale for combination of vaccination with anti-PD-1 treatment (Fig. 4F).

The C27a adjuvant combination was also evaluated by comparing the anti-tumor effect between C27a, CL401 and the commercial adjuvant AS01. Renca tumor-bearing mice were immunized with CL401/CoPoP/Nes2LR, AS01/Nes2LR and C27a/Nes2LR on day 1, 8, and 15. Five out of seven mice in the C27a group exhibited good response, significantly delaying the time that tumor size reached to 0.8 cm. Mice in CL401 and AS01 group barely showed response to treatment (Supplemental Fig. S8).

To assess the vaccine efficacy in an experimental metastasis model, we established a simple Renca tumor cell lung seeding or metastasis model based on intravenous injection of cancer cells, to examine efficacy of the C27a system. After injection of Renca cells on day 0, C27a/Nes2LR, PoP27a/Nes2LR, Poly:IC/Nes2LR were administered on day 3 and day 10. Lung images (Fig. 4G) and number of nodules (Fig. 4H) data showed that metastasis was clearly observed in untreated, Poly:IC and PoP27a group. Among them, about 400 nodules in untreated group were counted. 200 nodules were counted in Poly:IC and PoP27a group while almost no nodules was observed in the C27a group. Mice body weight decreased dramatically in untreated, Poly:IC and PoP27a group on day 22 (Fig. 4I).

### 2.6 Tolerance of C27a

**Fig. 5:**
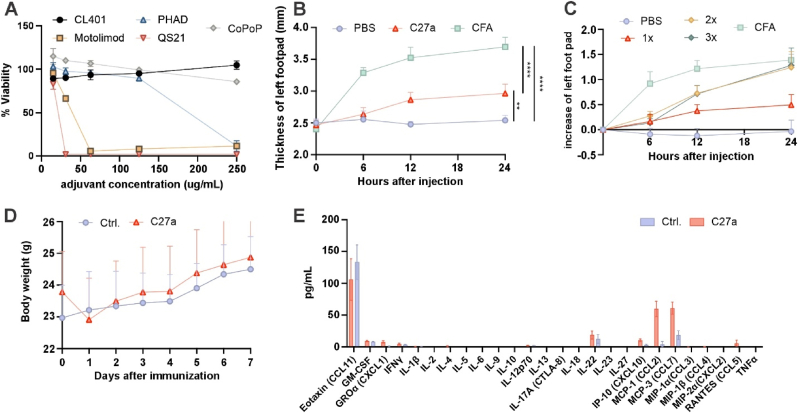
**C27a is well-tolerated to mice.A**, The viability of cells after treated with various concentrations of adjuvants used in C27a (including CL401, Motolimod, PHAD, QS-21 and CA liposomes). **B**, The thickness of left footpad after injection of PBS, 1x C27a (i.e, the standard dose) of Complete Freund’s adjuvant (CFA) at 0,6,12,24 h. **C**, The increase of left footpad after injected PBS, 1x/2x/3x C27a of CFA. The number multiples refer to the adjuvant dose, with 1x being the same C27a used in functional studies, and 2x being double and 3x being triple. **D**, the daily body weight following a single C27a vaccination. **E**, serum cytokine and chemokine secretion on day 2 after primary vaccination. Data was shown as mean ± SD (dots represent average of n = 3 (**A**), n = 5 (**B**), n = 4 (**C**) n = 6 (**D**), n = 4 (**E**) and analyzed by ordinary one-way ANOVA with Turkey’s multiple comparisons test. ∗*p* < 0.05, ∗∗*p* < 0.01, ∗∗∗*p* < 0.001, ∗∗∗∗*p* < 0.0001.

The impact of each component of C27a on cell viability was assess *in vitro*. Free QS-21 Motolimod and PHAD reduced cell viability nearly completely at concentrations of 31.25, 62.5, 250 μg/mL, respectively. In contrast, cells treated with CL401 or CoPoP maintained full cell viability in the concentration range assessed (Fig. 5A). Next, reactogenicity of C27a adjuvant system was assessed through measuring the local swelling induced in mouse footpads. Complete Freund’s Adjuvant (CFA), a well-known oil emulsion adjuvant that can induce strong immune response and inflammation was used as a benchmark. Following CFA injection, immediate and sustained footpad swelling persisted for 24 h. In contrast, the swelling induced by C27a appeared after 12 h and was significantly less than that caused by CFA (Fig. 5B). Even at 2× and 3× doses, local inflammation induced by C27a was comparable or less than CFA (Fig. 5C). For the *in vivo* toxicity study, mice received a single injection of C27a on day 0. A temporary decrease in body weight was observed on day 1, promptly recovering to levels comparable to the untreated group (Fig. 5D). Serum was collected 48 h after the primary vaccine for cytokine and chemokine assessment. The results showed that C27a did not cause any acute systemic upregulation of the tested cytokines. Increased levels of CCL2 and CCL7 may indicate an ongoing immune response (Fig. 5E). The complete blood count (CBC) results showed that almost all the values of 24 parameters tested were in the normal range when compared to untreated mice, indicating that at this dosage, C27a appears safe and well-tolerated to mice (Supplemental Fig. S9A). Additionally, no inflammatory immune cell infiltration, fibrosis or fibrin deposition was observed in the organ H&E staining sections of the vaccinated mice (Supplemental Fig. S9B).

## 3 Discussion

Adjuvants are key components for cancer vaccines to harness the innate immune system to enhance the adaptive one and in our case, elicit CD8^+^ T cells against specific tumor antigens. On the basis of the CoPoP system, we identified four adjuvants, including TLR 2/7, 4, 8 agonists and inflammasome, from 22 immunostimulants based on antigen-specific T cell production of IFN-γ which is an indicator of Th1 response. The earliest detection of antigen-specific CD8^+^ T cells in peripheral blood post-immunization occurs on day 5. During this period, exposure to the antigen is pivotal [37]. CL401 benefits early immune response due to the improved uptake in DCs and RAW264.7. The enhanced trafficking of liposomes to dLN facilitated by CL401 is consistent with previous reports that adjuvants can significantly boost the endocytosis of DCs for antigens, facilitating rapid presentation to T cells within a short period [38]. Similarly, formulated saponins have been shown to enhance antigen entry into dLN via promoting enhanced lymphatic drainage [39]. The combined use of multiple adjuvants may induce robust synergistic effects better than a single adjuvant.

### 3.1 The addition of further adjuvants to CL401 augmented later, but not earlier T cell elicitation

As shown in Fig. 1B, no other adjuvants screened alongside CL401 provided for improvements in the rapid eliciation neoantigen-specific T cells at the day 5 time point. However, by day 12, improvement in immunogenicity was observed with Motolimod, PHAD and QS-21, emphasizing the significance of diverse TLR signaling pathway combinations. This is synergy is consistent with other adjuvant studies. A PLGA based nanoparticle containing TLR4 and TLR7 agonists induced stronger humoral and T cell response [40]. Yellow fever vaccine YF-17D containing multiple TLR agonists synergistically regulated the secretion of proinflammation cytokines via activate the TLR ligands on APCs [41]. Activation of multiple TLR ligands can cause the cascade amplification of MyD88 mediator reflecting in high secretion levels of IL-1β, IL2, IL6, IL27 and TNFα in APCs, particularly IL-27 which is derived from IL12 family and promotes the differentiation of naïve CD4 T cells into Th1 cells. It shares the STAT1 signaling pathway with IFN-γ and exhibits functional overlap in mediating CTL responses [42]. This may also explain why CL401, as a dual agonist, can induce a rapid immune response at early stage. Notably, CL401 triggered limited IL-12p70 production in RAW264.7 and BMDC, while eliciting high levels of IL-10 specifically in BMDC. This phenomenon might be attributed to TLR2-mediated signaling, driving the immune response toward Th2 [43]. Different TLR agonists result in the different preference of Th response. The cytokine secretion profile indicated that C27a combination could induce mixed Th1/Th2 response. Simultaneously co-localized delivery of antigen and adjuvant is necessary to elicit an optimal immune response, which was also demonstrated in AS01 (QS-21 + TLR4 agonist MPLA) and AS04 (aluminum salt + TLR4 agonist MPLA) vaccines approved by FDA [25,44]. Meanwhile, the good biocompatibility of liposomes as vector greatly improves the uptake of antigens by APCs.

Our *in vivo* screening approach identified adjuvants that worked effectively with CoPoP liposomes to elicit MHC-I neoepitope-specific cellular responses with a short peptide particle vaccine. The screening in this study used a fixed adjuvant dose to iteratively identify promising adjuvant candidates, and it is certainly possible that differing dosing, formulation methodologies, antigen type, type of elicited immune response, host species and other variables would yield different responses. For example, it is likely that other responses might be observed if adjuvant dosing was higher or lower, and different adjuvants likely have different tolerated doses. Indeed in Fig. 5A, we observed that CoPoP and CL401 had minimal toxicity in the conditions tested, whereas QS-21 Motolimod and PHAD reduced cell viability at the increasing concentrations of 31.25, 62.5, 250 μg/mL respectively. However, different cell types and conditions might have different sensitives and the formulation itself may impact toxicity. For instance, as a saponin, QS-21 can disrupt the integrity of cell membrane. The formulation of QS-21 with cholesterol has been demonstrated to effectively reduce the toxicity of QS-21. C27a was shown to be effective and generally safe in two mouse tumor models. However, the limitation of this model is that different species have discrepancy in ligand expression and immune response. Even TLR4 expression varies across mice, non-human primates and humans, and differences in its expression pattern and sensitivity to agonists can lead to varied immune responses [45]. Additional animal models are necessary to validate its effectiveness and provide insights applicable to humans. Additionally, multiple adjuvants formulated in vaccines brings promising prospects for therapeutic effects but also raises major concerns about safety. While monophosphoryl lipid A and QS-21 are components of the approved AS01 adjuvant, the other two adjuvants, CL401 and Motolimod, have not been approved for vaccines. Further studies are required to better elucidate the toxicity of combined adjuvants as well as the manufacturing and commercial logistics of incorporating 4 independent adjuvants.

## 4 Conclusion

Here, we demonstrated that iterative *in vivo* screening can be used to develop a potent adjuvanted lipid nanoparticle, C27a, effective for cancer vaccines. Screening iterations identified TLR2/7, 4, 8 agonists and an inflammasome agent to incorporate for potent responses. C27a enhanced the uptake by APCs and significantly induced the T cell immune response in mice. Moreover, C27a exhibited effective anti-tumor capacity and demonstrated promise for co-administration with immune checkpoint blockade, displaying remarkable capabilities in inhibiting lung metastasis. From a practical perspective, CoPoP based C27a adjuvant system provided a versatile platform for murine cancer research, allowing safe coupling with various antigens.

### 4.1 Materials

Adjuvants were purchased from the vendors shown in Table S1, and LogP value were calculated from molinspiration. H2-K^d^ restricted Nes2LR (HHH-AYTTQREEL), H2-L^d^ AH1-5C (HHH-SPSYCYHQF), H2-D^b^ restricted E7_49-57_ (HHH-RAHYNIVTF) and HiLyte 488-His tagged Nes2LR were synthesized by GenScript. 1,2-dioleoyl-sn-glycero-3phosphocholine (DOPC, Corden LP-R4-076), cholesterol (PhytoChol, Wilshire Technologies), CoPoP, PoP (made by lab). Polyinosinic:polycytidylic acid (poly (I:C)) was from Sigma (Cat# P1530). RPMI 1640 (Gibco), Mouse GM-CSF (Miltenyi Biotec, Cat# 130-094-043), Mouse IFN-γ Single-Color ELISPOT kit (ImmunoSpot, Cat# mIFNgp-2M/10), APC-anti mouse CD8a antibody (53-6.7, BioLegend), FITC-anti mouse CD4 antibody (GK1.5, BioLegend), Live and dead (invitrogen), APC-CD11c (N418, Biolegend), PE-F4/80 (BM8, Biolegend), FITC-anti mouse CD279(PD-1) (29F.1A12, Biolegend). Collagenase type II (Gibco), DNase I (Roche). Cytokine & Chemokine 26-Plex Mouse ProcartaPlex™ Panel 1 kit (Invitrogen, Cat# EPX260-26088-901), Bouin’s Fixative (Azer scientific), HEK-Blue hTLR2 cell (InvivoGen), Freund Complete Adjuvant (Thermo fisher, Cat# 77140), Alamar blue (Thermo fisher, Cat# DAL1025), AS01 (GSK, Cat# NDC 58160-823-11). AS01 was obtained from Shingrix (McKesson).

### 4.2 Methods for vaccine preparation and characterization

CoPoP and PoP lipid were synthesized as previously reported [30,34]. CoPoP alone (CA) liposomes were prepared through extrusion. Briefly, DOPC, cholesterol and CoPoP lipid were dissolved in hot ethanol at 20:5:1 mass ratio, then 4-fold volume of PBS was added to the solution and heated to 60 °C for 10 min. Once fully dissolved, the liposomes were sequentially extruded ten rounds through a series of three stacked polycarbonate membrane filters with sizes of 200 nm, 100 nm, and 80 nm, from top to bottom. Ethanol in system was exchanged to PBS using 10 kD dialysis bag overnight. The concentration of CoPoP in liposomes was determined by UV spectrum at around 630 nm wavelength. During the adjuvant screening phase, with the exception of saponin derivatives, which were dissolved in an aqueous solvent, all other lipid-phase immunostimulatory adjuvants were dissolved in ethanol at 5 mg/mL. His-tagged peptide and various adjuvants were subsequently added to the CoPoP alone (CA) liposomes solution at a CoPoP to adjuvant to peptide mass ratio of 10:1:1 and then diluted by 2.5-fold using PBS. The mixture was sonicated for 1 h and particle size was measured by dynamic light scatter. For the PoP liposomes, DOPC, cholesterol, PoP lipids were dissolved in hot ethanol at a 20:5:1 mass ratio. The subsequent steps were identical to those involved in creating the CA liposomes. Peptides or adjuvants were then incubated with PoP liposome at a 0.1:1 mass ratio for 1 h at room temperature. The preparation of PoP/CL401 or PoP27a was same as mentioned above. For the Poly:IC group, each mice were given 50 μg Poly:IC incubated with peptides.

### 4.3 Fluorescence quenching

Fluorescence quenching occurs when a fluorophore approaches a porphyrin, leading to the absorption of their energy in the excited state by the porphyrin. His-tagged-HiLyte 488-Nes2LR was incubated with various liposome formulations at a peptide-to-DOPC mass ratio of 1:10 for 3 h at room temperature. PBS was the control group and every group had the triplicate wells. Subsequently, the samples were transferred to 250 μL PBS and excited with a 490 nm wavelength, while the fluorescence intensity was recorded at an emission wavelength of 527 nm. The binding rate was calculated as the following equation:

$$\%binding=\left[1-\frac{fluorescence\;intensity\left(experimental\;group\right)}{fluorescence\;intensity\left(control\;group\right)}\right]\times 100$$

### 4.4 Enzyme-linked immunosorbent spot (ELIspot) assay

Mouse blood samples were analyzed using a mouse IFN-γ single-color Elispot kit following the manufacturer’s instructions. Each BALB/c mouse was immunized with 50 μL various vaccines containing 2 μg antigens on day 0 and day 7. Blood was collected on day 5 and day 12 and proceed with red blood cell lysis buffer for 5 min 3 × 10^5^ cells were seed in each well of anti-IFN-γ antibody pre-coated 96-well plates and stimulated with 10 μg/mL peptides for 18–24 h at 37 °C incubator with 5 % CO_2_. The images were scanned and counted CTL ImmunoSpot S6 FluoroCore analyzer.

### 4.5 Flow cytometry analysis

Tetramer staining was used to analyze the population of antigen-specific CD8^+^ T cells. Both Nes2LR tetramer and AH1 tetramer were obtained from NIH Tetramer core facility and conjugated with PE. Mice were vaccinated on day 0 and day 7. 60 μL blood was collected from each mouse on day 7 and day 14 and incubated with 10 μL PE-tetramer (final 500× dilution) for 1 h at 4 °C. 30 μL Live and dead (final 500× dilution), APC-anti mouse CD8 antibody (final 200× dilution) and FITC-anti mouse CD4 antibody (final 200× dilution) were incubated with cells for 30 min. Cells were treated with 2 mL red blood cell lysis buffer for 5 min on ice, washed twice and resuspend in 200 μL FACS buffer (sterile DPBS with 0.5 % BSA and 0.01 % sodium azide) for flow cytometry.

For PD-1 expression study, 1 × 10^6^ splenocytes and tumor cells were incubated with PE-Nes2LR tetramer (final 500× dilution) for 1 h at 4 °C. Then live and dead (final 500× dilution), APC-anti mouse CD8 antibody (final 200× dilution) and FITC-anti mouse PD1 antibody (final 200× dilution) were incubated with cells for 30 min. Cells were washed twice and resuspend in 200 μL FACS buffer for analysis. FlowJo was used for data analysis.

### 4.6 HEK-blue hTLR2 reporter assay

HEK-Blue hTLR2 cells were cultured according to the instruction from InvivoGen. Briefly, cells were maintained in the DMEM supplemented with 10 % Fetal bovine serum (FBS), 1 % Pen-Strep (PS), 2 mM L-glutamine, 4.5 g/L glucose, 100 μg/mL Normocin and 1x HEK-Blue selection. CL401, Motolimod, PHAD and QS-21 were incubated with CoPoP liposome, individually, as described previously. All the four adjuvants and CoPoP liposomes were incubated together to make C27a. Free CL401 was dissolved in the physiological water. 20 μL 10 μg/mL samples were incubated with 5 × 10^4^ cells for 16 h. SEAP was read at 650 nm wavelength.

### 4.7 Vaccine uptake *in vivo* and *in vitro* assay

To evaluate the uptake of vaccines *in vivo*, inguinal lymph nodes of BALB/c mice, on the same side as the intramuscular injection, were harvested 24 h after administrating PoP and PoP/CL401 (containing 2 μg CL401) and smashed into single cell suspension using 100 μm cell strainer. Cells were incubated with 50 μL Live and dead (final 500× dilution), APC-CD11c antibody (final 200× dilution) and PE-F4/80 antibody (final 200× dilution) for 30 min at 4 °C and washed once for flow cytometry analysis. For the fluorescence images, after the same administration regimen, inguinal lymph nodes were cryo-sectioned into 10 μm slides and sealed using antifade mounting medium with DAPI.

The PoP signal was observed by EVOS FL Auto. For the *in vitro* uptake, 5 × 10^4^ RAW264.7 cells per well were seeded in 96-well plate and incubated with 100 μg/mL PoP and PoP/CL401 for 3 h at 37 °C. The PoP signal was visualized by EVOS FL Auto. Then, all the cells were lysed and the fluorescence of PoP was measured under excitation wavelength 490nm/emission wavelength 527 nm.

### 4.8 Mouse bone marrow-derived dendritic cells *in vitro* generation

BALB/c mice femurs and tibias were rinsed five times with PBS and a suspension of mouse bone marrow cells was obtained. Following passage through a 100 μm cell strainer, the single cell suspension was treated with red blood cell lysis buffer and incubated for 5 min on ice. The cells were then cultured in RPMI 1640 supplemented with 10 % (v/v) FBS, 1 % PS solution, 1 mM sodium pyruvate, 0.1 mM 2-mercaptoethanol, and 50 ng/mL GM-CSF at 37 °C with 5 % CO_2_, adjusted to a concentration of 2.5 × 10^6^ cells per mL. Half of the GM-CSF-containing media was replaced on days 3 and 6. Mouse bone marrow-derived dendritic cells (BMDCs) were harvested on day 7 for further analysis.

### 4.9 Cell culture

The Renca (CRL-2947) and RAW264.7 cell lines (TIB-71) were obtained from the American Type Culture Collection. TC-1 cells were the gift form Dr. Jorge Gomez-Gutierrez (University of Louisville, Kentucky, USA). Renca cells were maintained in RPMI 1640 supplemented with 10 % FBS, 1 % PS, 1 mM non-essential amino acids, 1 mM sodium pyruvate, 2 mM L-glutamine and have been passaged 12 times before inoculation. TC-1 cells were maintained in RPMI 1640 supplemented with 10 % FBS, 1 % PS and have been passaged 11 times before inoculation. RAW264.7 cells were maintained in DMEM supplemented with 10 % FBS, 1 % PS and the passage 24 was used for cell assay. All of them cultured at 37 °C with 5 % CO_2_. For the tumor microenvironment study, Renca tumors were resected from mice and digested in 5 mL DMEM containing 2 mg/mL collagenase type II and 100 μg/mL DNase I for 1 h at 37 °C. Tumor cells were dissociated into single cells through a 70 μm cell strainer.

### 4.10 Cell toxicity assay

4 × 10^4^ RAW264.7 cells were seed in 96 well plate overnight. Free QS-21, Motolimod, PHAD, QS-21 and CA liposomes were diluted in complete medium at 15.6, 31.25, 62.5, 125, 250 μg/mL 100 μL samples and media were incubated with RAW264.7 cells overnight. 10 μL Alamar blue was added to each well and incubated at 37 °C for 4 h. Plate was read at 560 nm excitation wavelength and 590 nm emission wavelength. Cell viability was calculated with the equation:

$$\%viability=\frac{fluorescence\;intensity\left(experimental\;group\right)}{fluorescence\;intensity\left(Media\right)}\times 100$$

### 4.11 Luminex cytokine multiplex assay

RAW264.7 and BMDCs were seeded in 96-well plates at a density of 5×10^4^ cells per well. After an overnight incubation, each well was replenished with fresh serum-free media. Subsequently, vaccines containing various adjuvants (0.625, 2.5, 10, 20 μg/mL final concentration of each adjuvant) was added and incubated. Cell culture supernatant was collected at 2, 4, 8 and 24 h. Blood samples obtained from immunized mice were lysed to remove red blood cells. 3×10^5^ cells plated in each well and stimulated by 10 μg/mL peptides in serum-free media overnight at 37 °C with 5 % CO_2_. Media as control was added to the untreated group. The supernatant was collected and analyzed using the Cytokine & Chemokine 26-Plex Mouse ProcartaPlex™ Panel 1 kit following the manufacturer’s instructions. The signal from Media group as the background was subtracted. All the groups were performed in triplicate. The standard curve range of each factor was shown in Table S2.

### 4.12 Murine study

5∼6-week-old female BALB/c and C57Bl/6 mice were obtained from Charles River Laboratories. All animal studies were conducted in compliance with the approved protocols of the Institutional Animal Care and Use Committee (IACUC) at the University at Buffalo. The liposome vaccines used in murine study were prepared through extrusion. For Renca tumor implantation, BALB/c mice were subcutaneously inoculated with 1× 10^5^ Renca cells at the right flank on day 0. Nes2LR neopeptide (5 μg per mouse) was incubated with C27a (containing 10 μg CL401, 2 μg Motolimod, 2 μg 3D6A-PHAD, 2 μg QS-21 per mouse), CL401/CoPoP (containing 10 μg CL401), AS01 (containing 2 μg MPLA and 2 μg QS-21) at room temperature for 1 h. Mice were immunized intramuscularly on day 1, 8 and 15. For the ICB combination therapy, when the tumor volume reached approximately 4 mm^3^, mice were intramuscularly immunized with C27a/Nes2LR vaccine on day 5, 12 and 17. Additionally, 100 μg of anti-PD-1 antibody per mouse was administered via intraperitoneal injection on day 7, 9, 14, and 16. For the TC-1 tumor implantation, C57Bl/6 mice were subcutaneously inoculated 1 × 10^5^ TC-1 cells at the right flank on day 0. When tumor volume reached to ∼80 mm^3^, mice were vaccinated with C27a/E7_49-57_ (5 μg E7_49-57_, 10 μg CL401, 2 μg Motolimod, 2 μg 3D6A-PHAD, 2 μg QS-21 per mouse) vaccine intramuscularly on day 9, day 16 and day 23. Tumor growth was monitored as required in protocol. Mice were euthanized when tumor volume (volume = length × width × width/2) reached the end point as well or if the tumor developed ulceration.

To establish the lung metastasis model, 1 × 10^5^ Renca cells were injected via the tail vein of BALB/c mice on day 0. Mice were untreated or treated with C27a/Nes2LR, PoP27a/Nes2LR (5 μg Nes2LR, 10 μg CL401, 2 μg Motolimod, 2 μg 3D6A-PHAD, 2 μg QS-21), Poly:IC/Nes2LR (5 μg Nes2LR, 50 μg Poly:IC) vaccines on day 3 and day 10. Body weight was monitored as required in protocol. Lungs were harvested on day 22. After washing by PBS, lung weights were recorded and stained in Bouin’s Fixative solution overnight. White nodules were counted.

### 4.13 Reactogenicity and CBC assay

The left footpads of mice were injected with 50 μL of PBS, 1x/2x/3x C27a, or CFA. The 1x C27a dose was the same as that used in the tumor study. CFA was prepared by mixing PBS and CFA in a 1:1 vol ratio. The thickness of the left footpad was measured using calipers at 0 (before injection), 6, 12 and 24 h post-injection. Body weight was measured every day after prime and serum was collected at 48 h for Luminex analysis and at day 7 for the complete blood count (CBC) and serum chemistry analysis.

### 4.14 Statistics

Statistical analyses were performed with GraphPad Prism. A test for synergism was performed using a linear contrast based on a fitted ANOVA model. Synergism in this context is defined as the difference between C27A and CA being greater in magnitude than the sum of the effects of CL401, Motolimod, PHAD, and QS-21 as compared to CA.

## CRediT authorship contribution statement

**Yuan Luo:** Investigation. **Shiqi Zhou:** Investigation. **Yiting Song:** Investigation. **Wei-Chiao Huang:** Investigation. **Gregory E. Wilding:** Investigation. **James Jablonski:** Investigation. **Breandan Quinn:** Investigation. **Jonathan F. Lovell:** Writing – original draft, Investigation.

## Ethics approval and consent to participate

This study did not involve any human participation.

All animal studies were carried out according to protocols approved by the University at Buffalo IACUC (protocol # BME13028Y).

## Declaration of competing interest

The authors declare the following financial interests/personal relationships which may be considered as potential competing interests: J.F.L. and W.-C. H. hold interest in POP Biotechnologies.
