# Supplementary material for: Iterative selection of lipid nanoparticle vaccine adjuvants for rapid elicitation of tumoricidal CD8⁺ T cells
Source: Bioact Mater. 2025 Feb 18;48:189–99. doi: 10.1016/j.bioactmat.2025.01.028 (PMC11880734; doi:10.1016/j.bioactmat.2025.01.028)
Supplement: Multimedia component 1 [file mmc1.docx]

**Supplemental information: Iterative Selection of Lipid Nanoparticle Vaccine Adjuvants for Rapid Elicitation of Tumoricidal CD8^+^ T cells**

Yuan Luo, Shiqi Zhou, Yiting Song, Wei-Chiao Huang, Gregory E. Wilding, James Jablonski, Breandan Quinn, Jonathan F Lovell


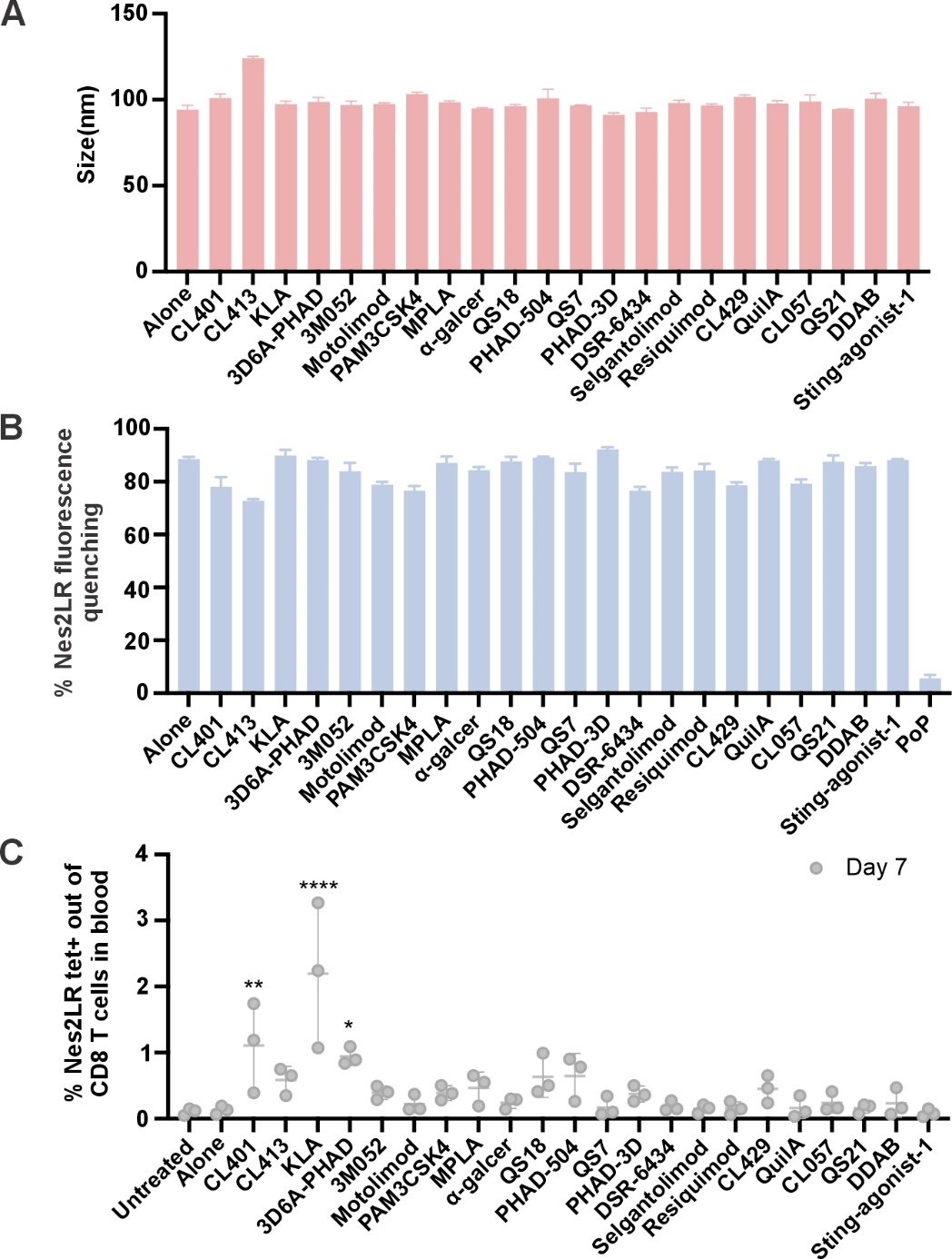


**Supplemental Figure S1. Size, binding and frequency of Nes2LR specific CD8 T cells of initial screen.** The size (**A**), fluorescence quenching of a fluorescently labeled peptide consistent with peptide binding (**B**) and population of Nes2LR specific CD8 T cells (**C**) coming from CoPoP/Nes2LR mixed with each single adjuvant. Data was shown as mean ± SD with n=3 and analyzed by ordinary one-way ANOVA with Dunnett’s multiple comparisons test. **p*<0.05, ***p*<0.01, ****p*<0.001, *****p*<0.0001.


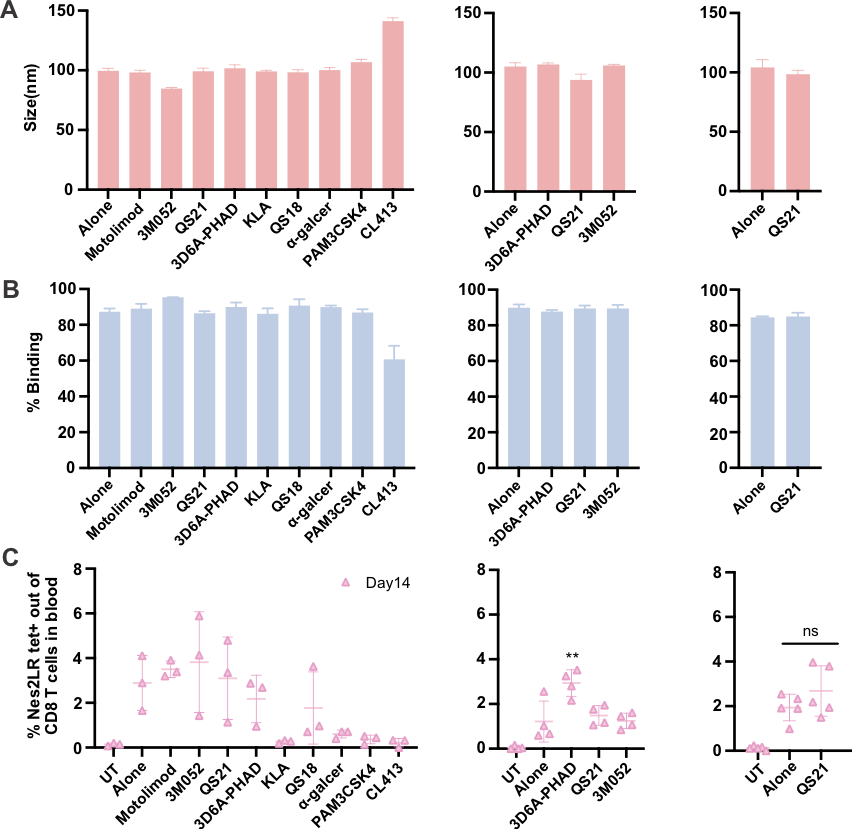


**Supplemental Figure S2. Size, binding and frequency of Nes2LR specific CD8 T cells of second, third and fourth.** The size (**A**), fluorescence quenching binding (**B**) and population of Nes2LR specific CD8 T cells (**C**) coming from CoPoP/Nes2LR/CL401, CoPoP/Nes2LR/CL401/Motolimod or CoPoP/Nes2LR/CL401/Motolimod/3D6A-PHAD mixed with each single adjuvant. Data was shown as mean ± SD with n=3, 4, 5 and analyzed by ordinary one-way ANOVA with Dunnett’s multiple comparisons test. **p*<0.05, ***p*<0.01, ****p*<0.001, *****p*<0.0001.


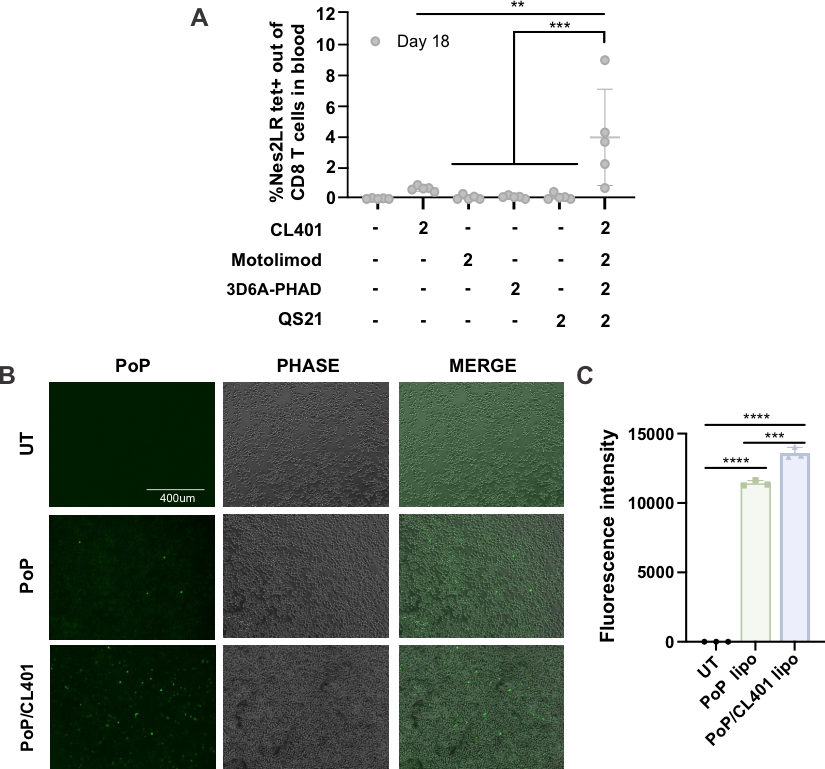


**Supplemental Figure S3. Improved immune response and C27a uptake by macrophages. A,** Nes2LR neoantigen tetramer staining**. B,** the fluorescence images of RAW264.7 incubated with media, 100 ug/mL PoP and PoP/CL401 liposomes for 3 hours. The green represented the fluorescence signal of PoP. **C,** quantitative analysis of PoP fluorescence in RAW264.7 cells. Data was shown as mean ± SD with n=5 (**A**), n=3 (**B, C**) and analyzed by ordinary one-way ANOVA with Dunnett’s multiple comparisons test. **p*<0.05, ***p*<0.01, ****p*<0.001, *****p*<0.0001.


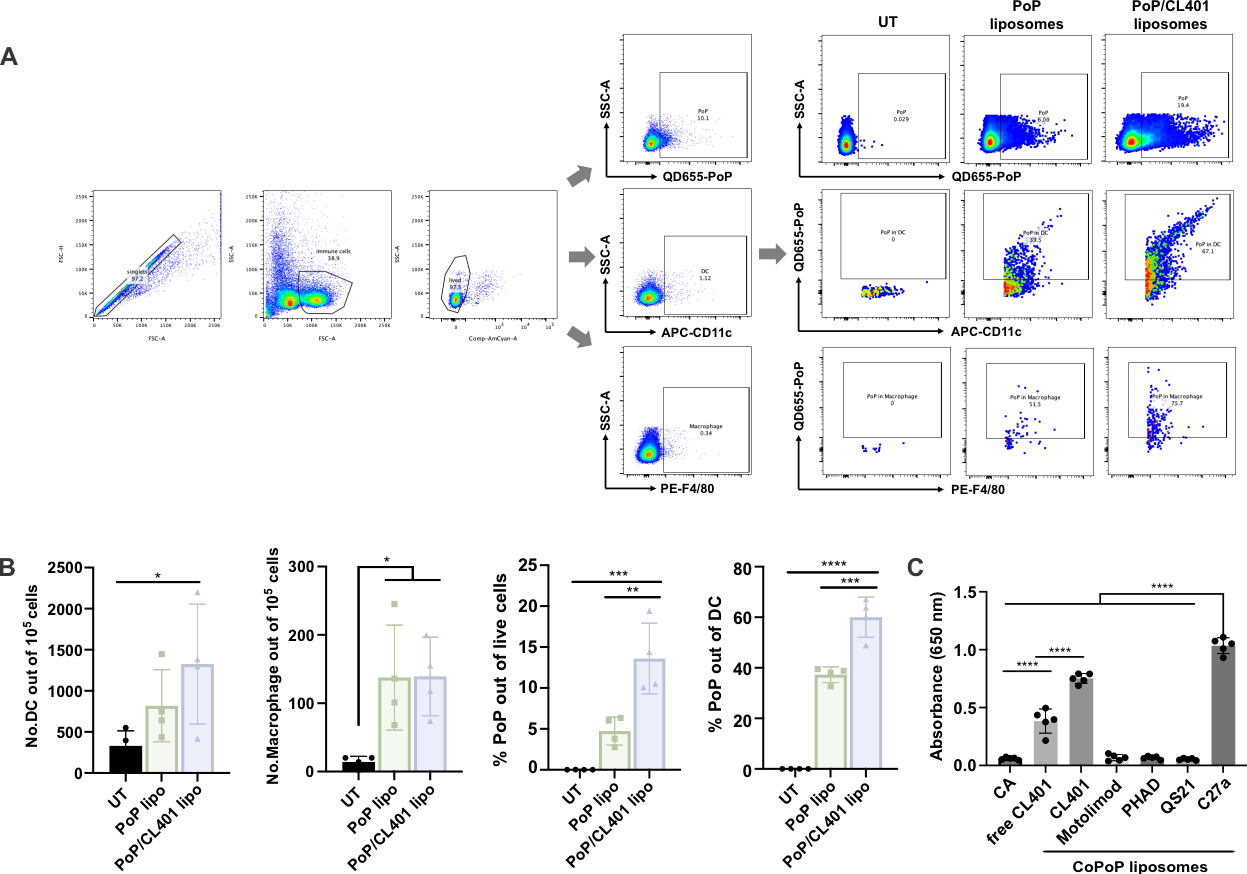


**Supplemental Figure S4. CL401 *in vivo* uptake and TLR2 signaling pathway. A,** the gating strategy of PoP in lymph nodes. Lived cells were gated out by singlets (FSC-A, FSC-H), cells (FSC-A, SSC-A), live and dead (AmCyan, SSC-A) successively. Then, the PoP, DCs and macrophages were gated out by QD655, APC (CD11c) and PE (F4/80). **B,** the number of DCs and macrophages out of 10^5^ live cells and the percentage of PoP positive cells among live cells and DCs in lymph nodes after 24 hours. **C,** SEAP detection from human HEK-blue-hTLR2 reporter cells. Cells were treated with CoPoP alone liposomes, free CL401, CL401/CoPoP liposomes, Motolimod/CoPoP liposomes, PHAD/CoPoP liposomes, QS21/CoPoP liposomes and C27a for 16 hours. Data was shown as mean ± SD with n=4 and analyzed by ordinary one-way ANOVA with Dunnett’s multiple comparisons test. **p*<0.05, ***p*<0.01, ****p*<0.001, *****p*<0.0001.


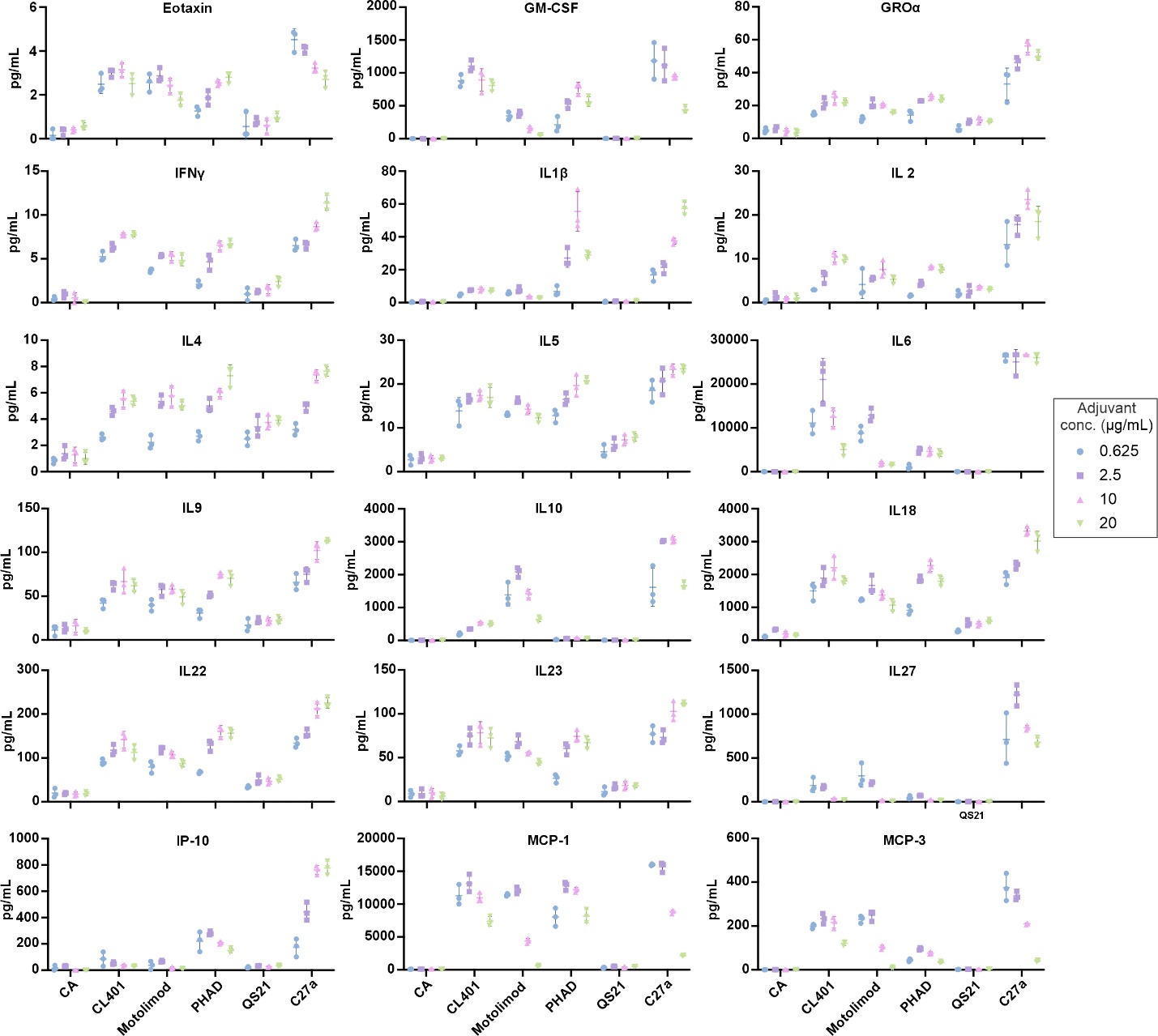


**Supplemental Figure S5. *In vitro* activity titration of adjuvants.** RAW264.7 cells were incubated with 0.625, 2.5, 10, 20 ug/mL CoPoP alone, CL401/CoPoP, Motolimod/CoPoP, PHAD/CoPoP, QS21/CoPoP and C27a liposomes 24 hours. Cell culture supernatant was collected for Luminex analysis.


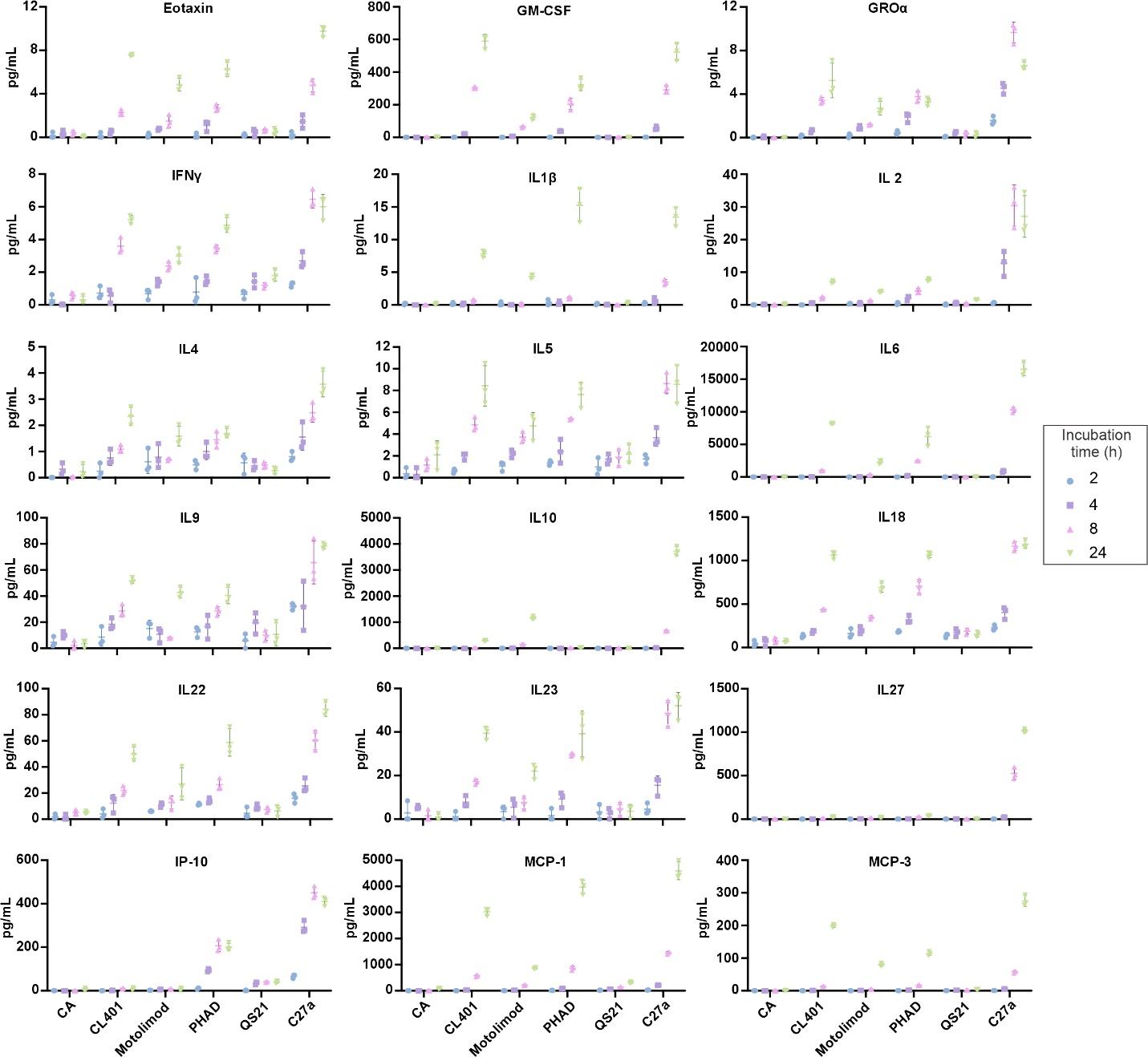


**Supplemental Figure S6. Time course of cytokine and chemokine secretion.** RAW264.7 cells were incubated with 10 ug/mL CoPoP alone, CL401/CoPoP, Motolimod/CoPoP, PHAD/CoPoP, QS21/CoPoP and C27a liposomes. Cell culture supernatant was collected at 2, 4, 8, 24 hours for Luminex analysis.


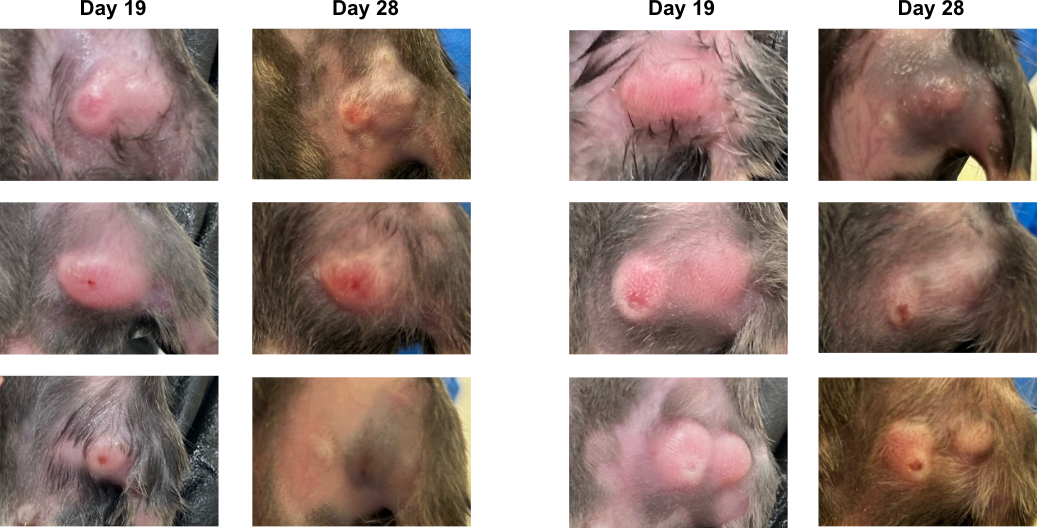


**Supplemental Figure S7. Images of TC-1 tumor volume during C27a vaccine treatment.** Mice were immunized C27a/E7_49-57_ vaccine on day 9, 16 and 23. Each tumor was record by photo at day 19 and day 28.


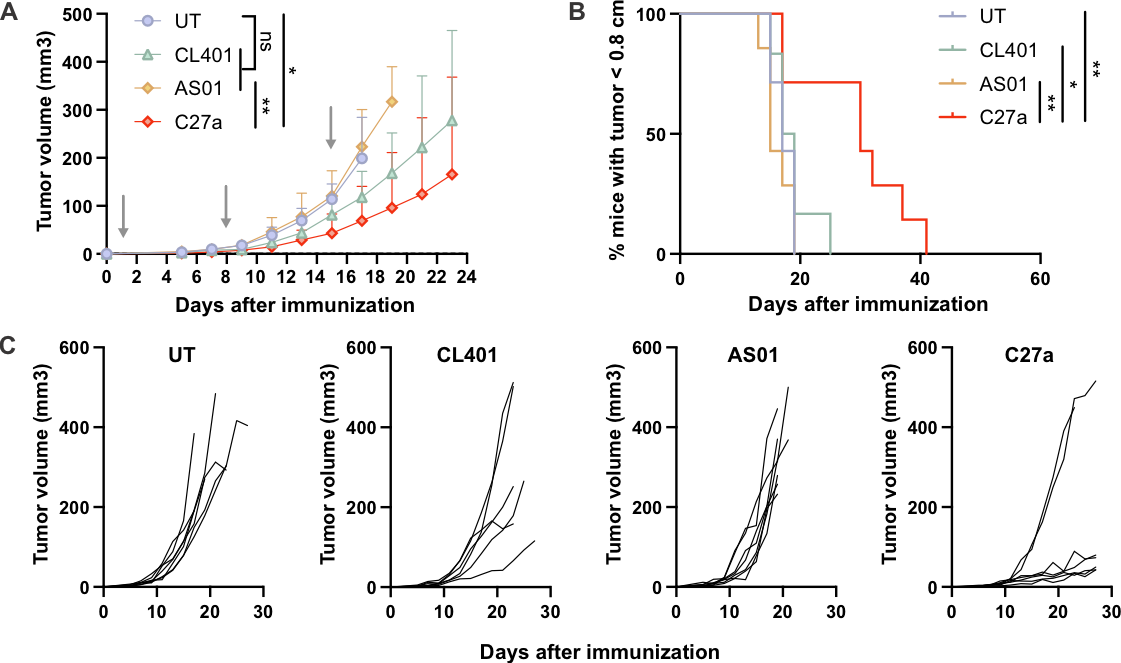


**Supplemental Figure S8. Anti-tumor effect of C27a, CL401 and AS01 vaccines. A,** Renca tumor growth curve. Mice were immunized CL401/CoPoP/Nes2LR, AS01/Nes2LR and C27a/Nes2L vaccine on day 1, 8, 15 (5 ug antigens per mouse). **B,** The percentage of mice bearing with Renca tumor long side < 0.8 cm. **C,** The individual tumor growth rate in each group. Data was shown as mean ± SD (A, n=6 for CL401, n=7 for others) and analyzed by ordinary one-way ANOVA with Turkey’s multiple comparisons test (A, the significance indicated the data on day 17) or Log-rank (Mantel-Cox) test (B). *p<0.05, **p<0.01.


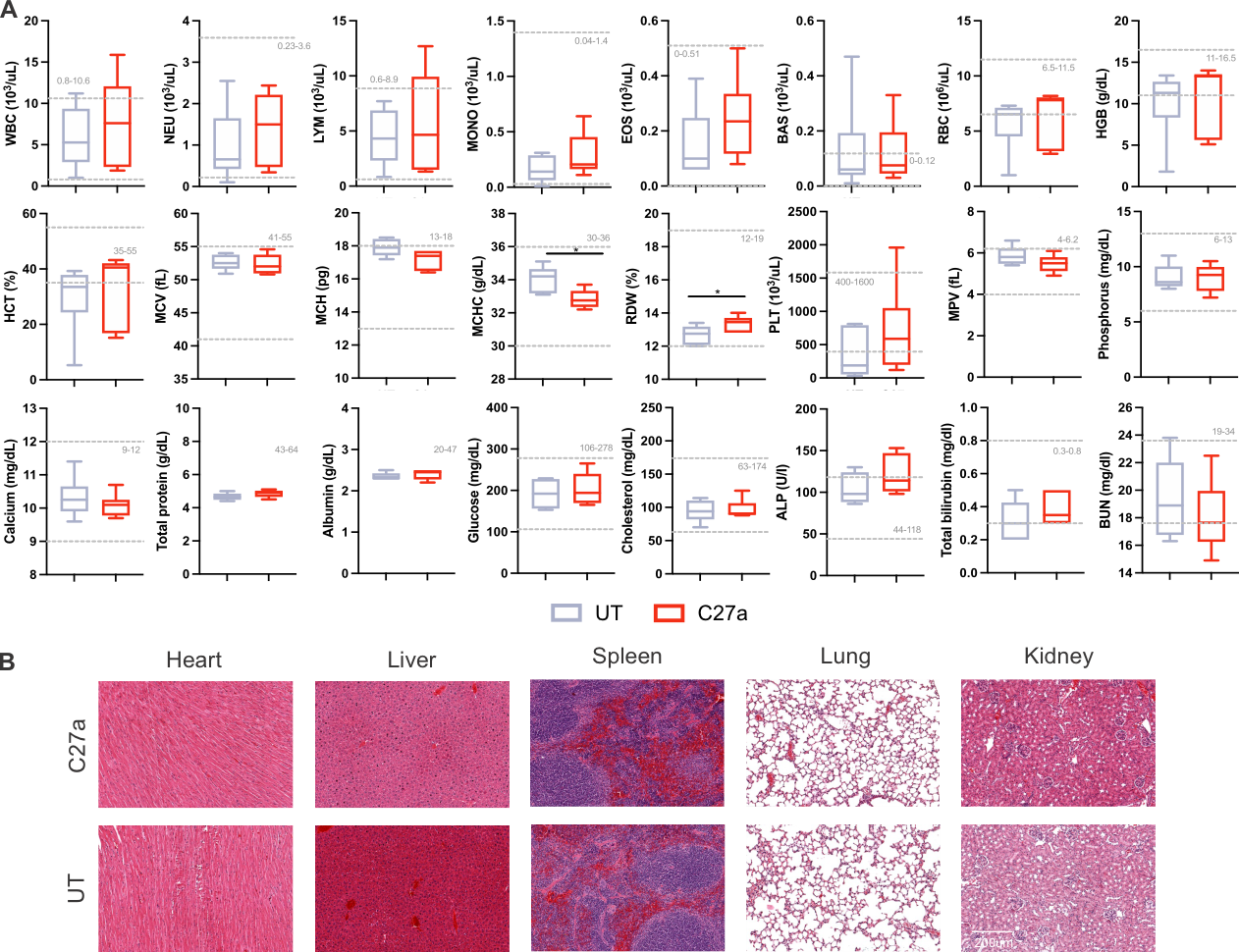


**Supplemental Figure S9. CBC and serum chemistry panel and H&E staining images. A,** Mice were injected a single dose of C27a on day 0 or were untreated (“UT”). Whole blood and serum were collected on day 7 for CBC and serum chemistry analysis. **B,** The HE staining images of heart, liver, spleen, lung and kidney. Mice were treated with or without C27a/Nes2LR vaccine twice and organs were harvested on day 14. Scale bar = 200 um. Magnification = 10x in Aperio ImageScope. Each group has triplicate. Data was shown as box plot with min and max (A=6) and analyzed by unpaired t test. **p*<0.05, ***p*<0.01, ****p*<0.001, *****p*<0.0001.

**Table S1. List of Screened Adjuvants**

| **Compound** | **Class** | **Milogp*** | **Vendor**** | **Putative lipid**  **particle binding** | **Solubility strategy** |
| --- | --- | --- | --- | --- | --- |
| QS18 | Inflammasome |  | Desert King | Cholesterol binding | Aqueous |
| QS21 | Inflammasome |  | Desert King | Cholesterol binding | Aqueous |
| QuilA | Inflammasome |  | Invivogen (vac-quil) | Cholesterol binding | Aqueous |
| QS7 | Inflammasome |  | Desert King | Cholesterol binding | Aqueous |
| DDAB | Cationic & alkyl tail |  | Avanti (890810) | Lipid-like | Ethanol |
| PAM3CSK4 | TLR1/2 |  | Invivogen (vac-pms) | Lipid-like | Ethanol |
| PAM2C-CL401 | TLR2/7 |  | Invivogen (vac-c401-5) | Lipid-like | Ethanol |
| PAM2C-CL413 | TLR2/7 |  | Invivogen (vac-c413-5) | Lipid-like | Ethanol |
| PAM2C-CL429 | TLR2/NOD2 |  | Invivogen (vac-c429) | Lipid-like | Ethanol |
| 3M052 | TLR7/8 | 9.47 | MedChemExpress (HY-109104) | Hydrophobic | Ethanol |
| Resiquimod (R848) | TLR7/8 | 2.15 | MedChemExpress (HY-13740) | Hydrophobic | Ethanol |
| Alpha-gal-cer | iNKT ligand | 10.12 | Avanti (867000) | Lipid-like | Ethanol |
| DSR-6434 | TLR7 | 2.77 | MedChemExpress (HY-110120) | Hydrophobic | Ethanol |
| 3M-002(CL075) | TLR8 | 3.2 | MedChemExpress (HY-117066 ) | Hydrophobic | Ethanol |
| Selgantolimod | TLR8 | 2.48 | MedChemExpress (HY-109137) | Hydrophobic | Ethanol |
| Motolimod | TLR8 | 4.2 | MedChemExpress (HY-13773) | Hydrophobic | Ethanol |
| STING-Agonist-1 | STING | 3.6 | MedChemExpress (HY-19711) | Hydrophobic | Ethanol |
| 3D6A-PHAD | TLR4 |  | Avanti (699855) | Lipid-like | Ethanol*** |
| MPLA | TLR4 |  | Avanti (699800) | Lipid-like | Ethanol |
| Kdo2-lipidA | TLR4 |  | Avanti (699500) | Lipid-like | Ethanol*** |
| PHAD-3D | TLR4 |  | Avanti (699852) | Lipid-like | Ethanol*** |
| PHAD-504 | TLR4 |  | Avanti (699810) | Lipid-like | Ethanol |

***Log P calculated from** Milogp <https://www.molinspiration.com/cgi-bin/properties>

** Catalog numbers are shown in parentheses where applicable

*** Some cloudiness was observed in ethanol solution

**Table S2. Mouse Cytokine and Chemokine Luminex standard curve**

|  | **Sensitivity (pg/mL)** | **Standard curve range (pg/mL)** |
| --- | --- | --- |
| Eotaxin | 0.01 | 0.5-2000 |
| GM-CSF | 0.19 | 2.4-10000 |
| Groα | 0.05 | 1.7-7000 |
| IFNγ | 0.09 | 1.2-5000 |
| IL1β | 0.14 | 1.2-5000 |
| IL2 | 0.1 | 1.2-5000 |
| IL4 | 0.03 | 1.2-5000 |
| IL5 | 0.32 | 2.4-10000 |
| IL6 | 0.21 | 4.9-20000 |
| IL9 | 0.28 | 18.3-75000 |
| IL10 | 0.69 | 2.4-10000 |
| IL12p70 | 0.21 | 2.4-10000 |
| IL13 | 0.16 | 2.4-10000 |
| IL17A | 0.08 | 1.2-5000 |
| IL18 | 9.95 | 36.6-150000 |
| IL22 | 0.24 | 12.2-50000 |
| IL23 | 2.21 | 12.2-50000 |
| IL27 | 0.34 | 2.4-10000 |
| IP-10 | 0.26 | 0.5-2000 |
| MCP-1 | 3.43 | 7.3-30000 |
| MCP-3 | 0.15 | 0.2-1000 |
| MIP-1α | 0.13 | 0.5-2000 |
| MIP-1β | 1.16 | 1.2-5000 |
| MIP-2 | 0.37 | 0.7-3000 |
| RANTES | 0.35 | 2.4-10000 |
| TNFα | 0.39 | 3.7-15000 |

**Table S3. secretion levels of cytokines and chemokines**


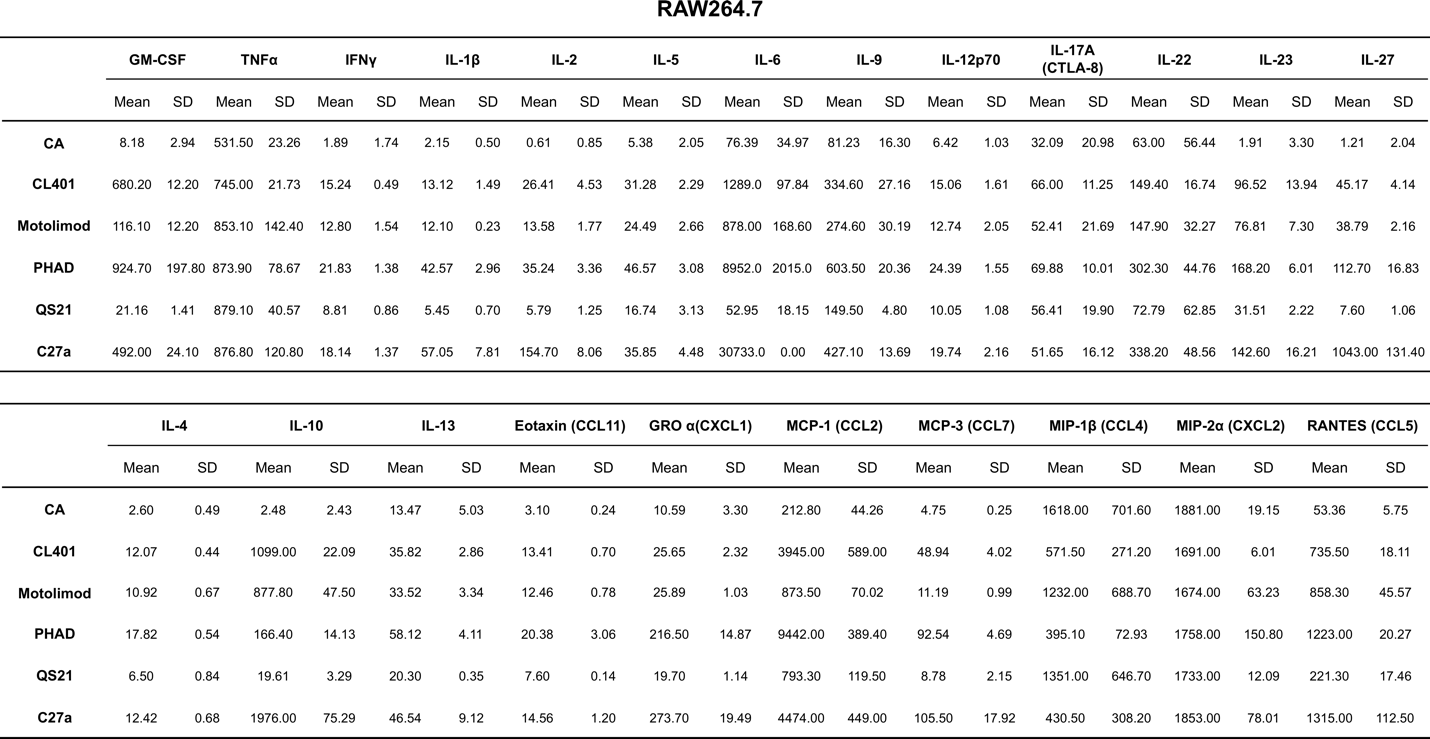


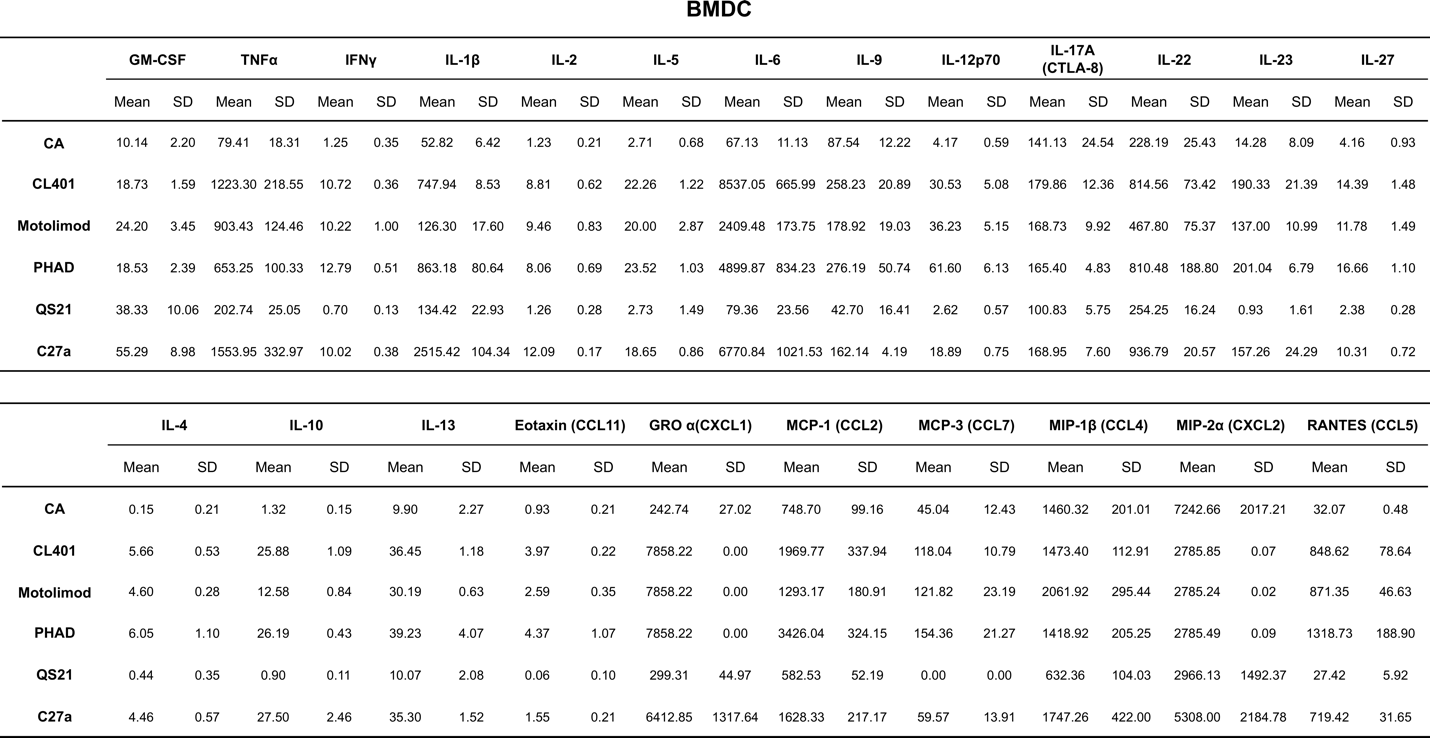


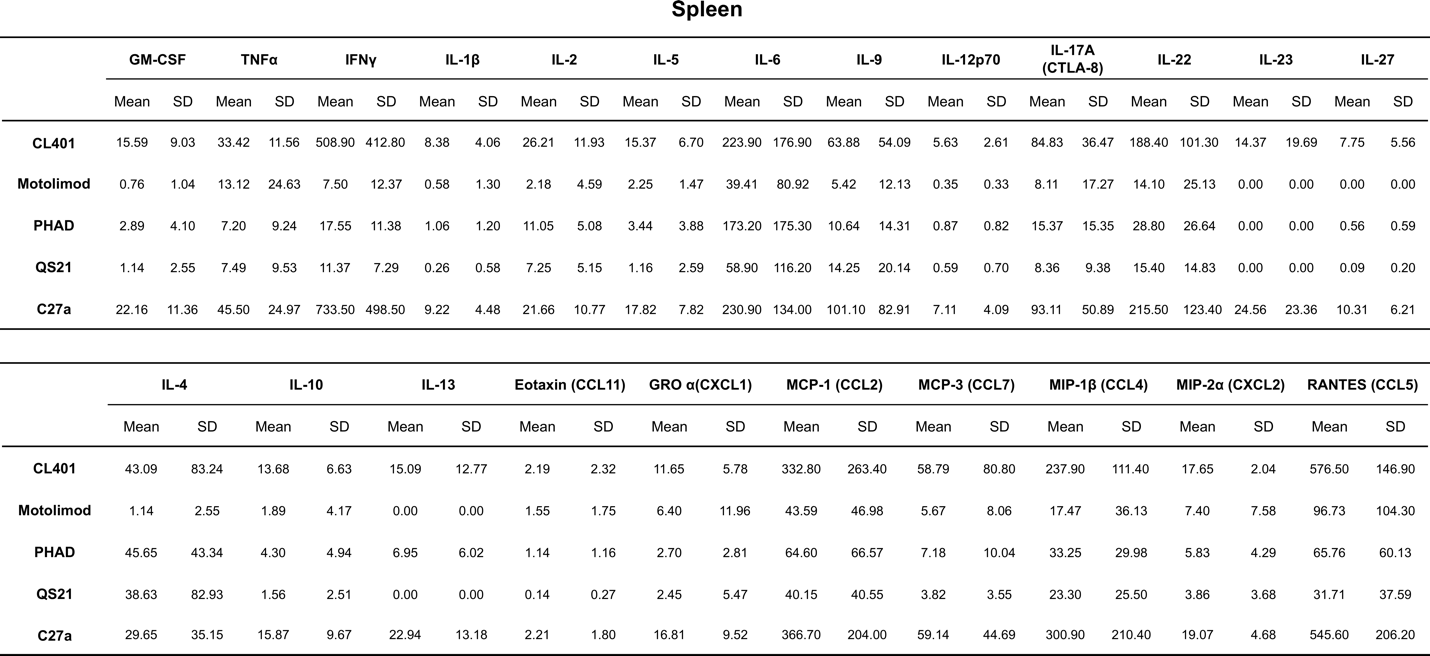


All concetration unit is pg/mL
